# Racial disparities in renal cell carcinoma: a single‐payer healthcare experience

**DOI:** 10.1002/cam4.755

**Published:** 2016-05-26

**Authors:** Abiodun Mafolasire, Xiaopan Yao, Cayce Nawaf, Alfredo Suarez‐Sarmiento, Wong‐Ho Chow, Wei Zhao, Douglas Corley, Jonathan N. Hofmann, Mark Purdue, Adebowale J. Adeniran, Brian Shuch

**Affiliations:** ^1^ Department of UrologyYale School of MedicineNew HavenConnecticut; ^2^ Yale Center for Analytical SciencesYale School of MedicineNew HavenConnecticut; ^3^ Department of EpidemiologyAnderson Cancer CenterHoustonTexas; ^4^ Kaiser Permanente Division of ResearchKaiser Permanente San Francisco Medical CenterSan FranciscoCalifornia; ^5^ Division of Cancer Epidemiology and GeneticsNational Cancer InstituteRockvilleMaryland; ^6^ Department of PathologyYale School of MedicineYale UniversityNew HeavenConnecticut

**Keywords:** Health disparity, kidney cancer, outcome, RCC, survival

## Abstract

Significant racial disparities in survival for renal cell carcinoma (RCC) exist between white and black patients. Differences in access to care and comorbidities are possible contributors. To investigate if racial disparities persist when controlling for access to care, we analyzed data from a single‐payer healthcare system. As part of a case–control study within the Kaiser Permanente Northern California system, pathologic and clinical records were obtained for RCC cases (2152 white, 293 black) diagnosed from 1998 to 2008. Patient demographics, comorbidities, tumor characteristics, and treatment status were compared. Overall survival and disease‐specific survival (DSS) were calculated by the Kaplan–Meier method. A Cox proportion hazards model estimated the independent associations of race, comorbidity, and clinicopathologic variables with DSS. We found that compared to white patients, black patients were diagnosed at a younger age (median 62 vs. 66 years, *P *<* *0.001), were more likely to have papillary RCC (15% vs. 5.2%, *P* < 0.001), and had similar rates of surgical treatment (78.8% vs. 77.9%, *P* = 0.764). On multivariate analysis, advanced American Joint Committee on Cancer (AJCC) stage, lack of surgical treatment, larger tumor size, and higher grade were predictors of worse DSS. Race was not an independent predictor of survival. Therefore, we conclude that within a single healthcare system, differences in characteristics of black and white patients with RCC persist; black patients had different comorbidities, were younger, and had decreased tumor stage. However, unlike other series, race was not an independent predictor of DSS, suggesting that survival differences in large registries may result from barriers to healthcare access and/or comorbidity rather than disease biology.

## Introduction

Over the last few decades, the incidence of renal cell carcinoma (RCC) has been increasing among all racial groups in the United States, with the largest increase occurring among black patients. Recently, the incidence of kidney cancer in black patients surpassed that of any racial group 1. Studies have shown various epidemiologic risk factors predisposing for RCC including male gender, chronic kidney disease (CKD), smoking, hypertension, and excess body mass index (BMI) 2, 3, 4, 5, 6. Many of these risk factors differ among racial groups, contributing to the differences in the epidemiologic trends. It also has been demonstrated that race influences the distribution of histologic subtypes, with black patients having increased frequency of papillary kidney cancer 3, 7. While black patients may present at an earlier disease stage 8, 9, 10, 11, paradoxically, survival appears to be worse 10, 12. Even when controlling for treatment and various prognostic factors such as stage, tumor size, and grade, a disparity in relative survival still persists between black and white patients with kidney cancer 12.

Racial differences in survival raise the question whether specific races have more aggressive forms of kidney cancer. Unfortunately in the United States, access to healthcare significantly differs by race 13, which may largely account for observed cancer‐related outcomes due to treatment barriers. Besides possible differences in tumor biology, this could account for the apparent worse survival in black patients with RCC. Data from National Cancer Institute's Surveillance Epidemiology and End Results (SEER) program demonstrates that black patients are less likely to undergo definitive surgical therapy (nephrectomy) 14, 15, 16. The decreased rate of nephrectomy may be influenced by greater preexisting comorbidities in black patients^14^. However, even when controlling for treatment, other studies have found black patients have worse outcome 12.

Analyses of survival outcomes using large registries may be unable to account for barriers to care and lead to an improper conclusion that those survival disparities are related to tumor biology. Studies in pediatric cancers have shown that differences in cancer‐related outcomes disappear when evaluated within a single healthcare system 17; however, in kidney cancer, there have been no studies evaluating racial disparities with similar methodology. By using a single‐healthcare payer database, we evaluate whether racial survival differences exist while controlling for demographics, tumor characteristics, comorbidities, and treatment in a system where all patients should receive equivalent cancer treatment.

## Materials and Methods

### Study design

To evaluate racial disparities between non‐hispanic white and black patients in an integrated‐managed care system, we conducted an investigation among RCC cases from a nested case–control study in the Kaiser Permanente Northern California (KPNC) health system 6. Patients receiving care from KPNC were included in our study if they had been diagnosed with RCC (International Classification of Diseases for Oncology, third edition, site code C64.9) between 1998 and 2008. The demographic data were obtained from membership databases which include age, race/ethnicity (self‐described), and sex. The cancer data were obtained from the Kaiser Permanente Cancer Registry, which reports directly to the Surveillance, Epidemiology, and End Results program. Cancer location, tumor size, T stage, grade, and AJCC stage, histology, treatment data, and survival are recorded for the registry using chart review. Comorbidity data (BMI, smoking history, hypertension, diabetes, CKD, any medical renal disease, and anemia) were defined using international classification of disease (version 9) coding associated with visits. Tumor AJCC stage was recoded in accordance with current seventh edition guidelines 18. Vital status and the last follow‐up date were available until December 31, 2013. The cause and date of death were assigned using a combination of cancer registry data and a mortality registry that concatenates state and federal mortality files, including primary cause of death from California death certificates.

### Statistical analysis

All analyses were performed using SAS 9.3 (SAS Institute, Inc., Cary, NC) and R version 3.1.2 (R Foundation for Statistical Computing, Vienna, Austria). For patient clinical characteristics, continuous variables were presented as median (range) and categorical ones as frequency with relative percentage. Group comparisons between black and white patients were performed with the use of Fisher's exact test or chi‐squared tests for categorical variables and Mann–Whitney *U* tests for continuous variables, as appropriate.

Overall survival (OS) was measured from the date of diagnosis to the date of death (based on the death certificate), alive patients were censored at the date of last follow‐up date. Disease‐specific survival (DSS) was measured from the date of diagnosis to the date of death caused by the disease, both alive patients and patients died due to other reasons censored at the last follow‐up date and death date, respectively. The association of survival with clinical and prognostic factors was tested using the log‐rank test. Prognostic factors that were significantly associated with DSS survival on univariate analysis and clinically relevant factors were included in a Cox proportional hazards model for multivariate analysis. Statistical significance was considered for *P* ≤ 0.05.

## Results

### Patient and disease characteristics

A total of 2445 patients were included, of which, 2152 (88.0%) were white and 293 (12.0%) were black. The median age of diagnosis for black patients was significantly lower than for white patients (62 years vs. 66 years, respectively, *P* < 0.001) (Table 1). Compared to white patients, a higher proportion of black patients had a history of hypertension (64.9% vs. 50.1%, *P *<* *0.001), CKD (10.2% vs. 0.7%, *P *= <0.001), and anemia (10.2% vs. 0.7%, *P *= <0.001). There were no significant racial differences in BMI, diabetes, or smoking history.

**Table 1:** Demographic, clinical, pathologic, outcomes data of white and black patients with RCC who received care with Kaiser Permanente Northern California from 1998 to 2008

Variable	Black patients (*N* = 293)	White patients (*N* = 2152)	*P*‐value
Age at diagnosis (years)			<.001
Median	62	66	
Range	31–91	26–92	
Gender			0.007
Male	166 (56.7%)	1392 (64.7%)	
Female	127 (43.3%)	760 (35.3%)	
BMI^a^			0.553
<25	9 (15%)	95 (20.8%)	
25–30	24 (40%)	163 (35.7%)	
>30	27 (45%)	199 (43.5%)	
Smoking history			0.263
Yes	81 (27.65%)	530 (24.6%)	
No	212 (72.4%)	1622 (75.4%)	
Hypertension			<0.001
Yes	190 (64.9%)	1077 (50.1%)	
No	103 (35.2%)	1075 (49.9%)	
Diabetes			0.094
Yes	58 (19.8%)	343 (15.9%)	
No	235 (80.2%)	1809 (84.1%)	
Chronic kidney disease			<0.001
Yes	30 (10.2%)	15 (0.7%)	
No	263 (89.8%)	2137 (99.3%)	
Renal disease			<0.001
Yes	35 (11.9%)	49 (2.3%)	
No	258 (88.1%)	2103 (97.7%)	
Anemia			<0.001
Yes	52 (17.7%)	181 (8.4%)	
No	241 (82.3%)	1971 (91.6%)	
Tumor size			0.066
Median	4.4	5.1	
Histologic subtype			<0.001
Clear cell	208 (71.0%)	1829 (85.0%)	
Papillary	44 (15.0%)	111 (5.2%)	
Chromophobe	12 (4.1%)	56 (2.6%)	
Others	29(9.9%)	156 (7.3%)	
Tumor grade^b^			0.463
Grade 1	30 (15.2%)	198 (14.2%)	
Grade 2	98 (49.7%)	648 (46.4)	
Grade 3	59 (29.9%)	441 (31.5%)	
Grade 4	10 (5.1%)	111 (7.9%)	
T stage^b^			0.053
T1/T2	215 (77.6%)	1424 (72.1%)	
T3/T4	62 (22.4%)	551 (27.9%)	
N stage^b^			0.640
N0/Nx	284 (96.9%)	2095 (97.4%)	
N+	9 (3.1%)	56 (2.6%)	
M stage			0.012
M0/Mx	247 (84.3%)	1677 (77.9%)	
M+	46 (15.7%)	475 (22.1%)	
AJCC staging^b^			0.008
Stage I/II	205 (70.2%)	1330 (62.2%)	
Stage III/IV	87 (29.8%)	808 (37.8%)	
Treatment status			0.723
Surgery	231 (78.8%)	1677 (77.9%)	
No surgery	62 (21.2%)	475 (22.1%)	
Vital status			0.202
Alive	152 (51.9%)	1031 (47.9%)	
Dead	141 (48.1%)	1121 (52.1%)	
Death from RCC			0.033
Alive	152 (51.9%)	1031 (47.9%)	
Dead from other cause	64 (21.8%)	626 (29.1%)	
Dead from RCC	77 (26.3%)	495 (23.0%)	

a BMI data only available for patients enrolled from 2005 to 2008.

b Missing variables include tumor grade (850 cases), T stage (193 cases), N Stage (one case), AJCC Stage (15 missing).

BMI, body mass index; RCC, renal cell carcinoma.

Tumor characteristics significantly differed between white and black patients. Papillary histology was more frequent in black patients than in white patients (15.0% vs. 5.1%, *P *= <0.001). At diagnosis, black patients presented less frequently with metastasis (15.7% vs. 22.1%, *P *=* *0.012) and had a greater frequency of localized disease (AJCC stage I/II) (70.2% vs. 62.2%, *P *=* *0.008). Although, not reaching statistical significance, black patients tended to have smaller sized tumors (4.4 cm vs. 5.1 cm, *P *=* *0.066). No racial differences were observed in tumor grade and nodal status. No statistical difference was found in the rate of surgical treatment between white and black patients (77.9% vs. 78.8%, *P *=* *0.764).

In patients with clear cell subtype, black patients were diagnosed at a younger median age (63 vs. 66, *P *=* *0.003). Black patients with clear cell RCC were less likely to be male (54.3% vs. 63.8%, *P *=* *0.008) (Table S2). In patients with papillary subtype, the median age at diagnosis for black patients was similarly younger compared to white patients (57.5 vs. 64, *P *=* *0.003). (Table S3).

**Figure 1:**
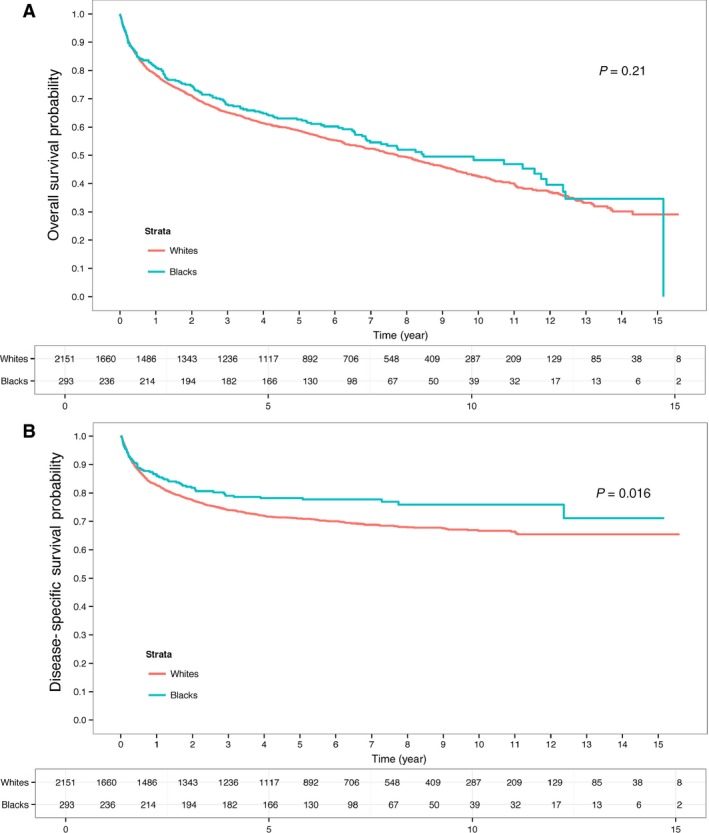
(A) Overall survival (OS) and (B) disease‐specific survival (DSS) for black and white patients diagnosed with kidney cancer who received treatment within Northern California Kaiser Permanente between 1998 and 2008.

RCC deaths were more frequent in black patients compared to white patients (26.3% vs. 23.0%, *P *=* *0.033). Median follow‐up time in all the surviving patients was 7.24 years. In the univariate analysis, there is no significant difference in terms of overall survival between black patients and white patients (*P *=* *0.21) (Fig. 1A). The median OS time for black patients is 8.47 year with 95% confidence interval (6.86, 11.75) and the median OS for white patients is 7.74 year with 95% confidence interval (7.09, 8.43). Multivariate analysis of OS demonstrated that advanced age, male gender, renal disease, larger tumor size, higher tumor grade and stage, and no surgical treatment were predictors of worse outcome (Table 2.) Race was also not found to be an independent predictor of OS (*P* = 0.463).

**Table 2:** Multivariate analysis of prognostic variables for overall specific survival (OS) in patients with kidney cancer who received care with Northern California Kaiser Permanente general from 1998 to 2008

Variable	Hazard ratio (HR)	95% CI	*P‐*value
Age	1.038		<0.001
Gender			0.0424
Male	1.00 (ref)		
Female	0.84	0.71–0.99	
Race			0.463
White	1.00 (ref)		
Black	1.101	0.85–1.42	
Renal Disease	2.31	1.60–3.34	<0.001
Tumor size (cm)	1.04	1.03–1.05	<0.001
Tumor grade			<0.001
Grade 1 and 2	1.00 (ref)		
Grade 3	1.19	1.00–1.41	0.051
Grade 4	2.27	1.76–2.93	<0.001
Histological subtype			0.292
Clear cell	1.00 (ref)		
Papillary	0.72	0.64–1.28	0.567
Others	0.824	0.64–1.06	0.132
AJCC stage			<0.001
Stage 1 and 2	1.00 (ref)		
Stage 3 and 4	2.597	2.21–3.05	
Treatment status			<0.001
Surgery	1.00 (ref)		
No surgery	4.241	3.295–5.458	

**Figure 2:**
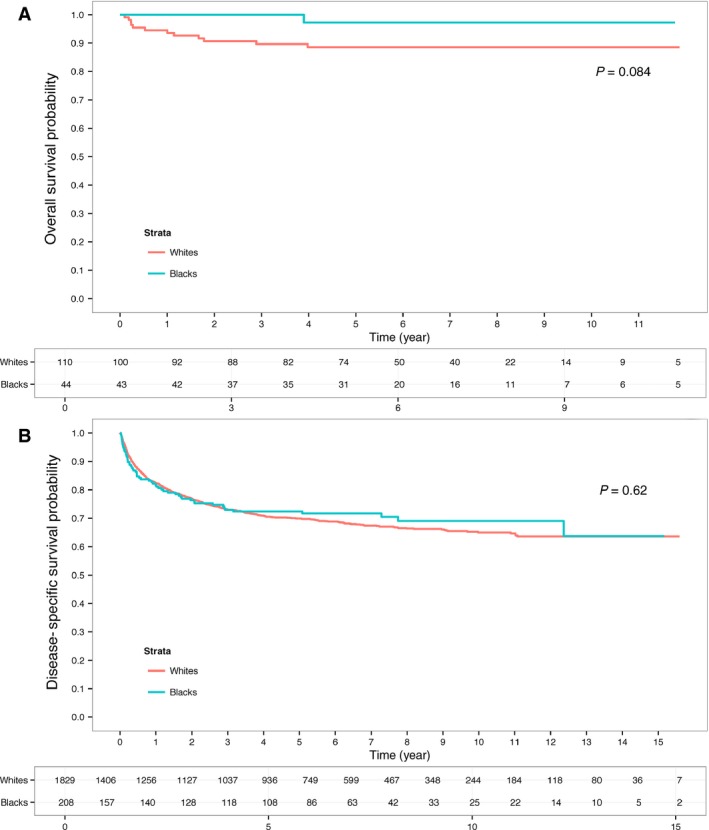
Disease‐specific survival (DSS) for black and white patients diagnosed with (A) Papillary and (B) Clear cell who received treatment within Northern California Kaiser Permanente between 1998 and 2008.

Surprisingly, black patients were found to have improved DSS (*P *=* *0.016) (Fig. 1B). The 5‐ and 10‐year DSS estimates for black patients versus white patients were 78% and 75% versus 71% and 67%, respectively. In univariate analysis, race, age, AJCC stage, grade, tumor size, histology, and no surgical treatment were found to be predictors of DSS (Table S1). In multivariate analysis, only larger tumor size, higher tumor grade, advanced AJCC stage and not being treated with surgery were found to be independent predictors of worse outcome. Age, histology, and race were no longer associated with DSS (Table 3). DSS was also evaluated by specific histology. For clear cell RCC, DSS was nearly identical (Fig. 2A) with a 5‐year survival of 72% versus 70% for black patients versus white patients, respectively (*P *=* *0.619). For papillary RCC, there was a trend for improved DSS for black patients with 5‐year survival estimate of 92% versus 78% (*P *=* *0.084) (Fig. 2B).

**Table 3:** Multivariate analysis of prognostic variables for disease specific survival (DSS) in patients with kidney cancer who received care with Northern California Kaiser Permanente General from 1998 to 2008

Variable	Hazard ratio (HR)	95% CI	*P‐*value
Age	1.001		0.896
Gender			0.964
Male	1.00 (ref)		
Female	0.99	0.78–1.27	
Race			0.382
White	1.00 (ref)		
Black	0.82	0.52–1.29	
Renal disease	1.43	0.52–3.94	0.481
Tumor size (cm)	1.05	1.04–1.06	<0.001
Tumor grade			<0.001
Grade 1 and 2	1.00 (ref)		
Grade 3	1.86	1.42–2.43	<0.001
Grade 4	3.32	2.36–4.69	<0.001
Histological subtype			0.364
Clear cell	1.00 (ref)		
Papillary	0.72	0.35–1.49	0.378
Others	0.809	0.56–1.15	0.241
AJCC stage			<0.001
Stage 1 and 2	1.00 (ref)		
Stage 3 and 4	8.63	6.48–11.5	
Treatment status			<0.001
Surgery	1.00 (ref)		
No surgery	5.74	4.23–7.81	

## Discussion

Using a large single‐healthcare system database, we examined the clinicopathologic characteristics and survival outcomes for black and white patients with kidney cancer. In this single healthcare system, clinical practice guidelines exist to ensure patients receive equal access to care and appropriate guideline‐based treatment. As expected in such a system, surgical treatment rates were similar across racial groups unlike what has been observed in population‐based and other multicenter registries. Much of the care included in such an organization is driven by evidence‐based, practice guidelines, something that may help to limit the variability in cancer treatment.

In our analysis, race was not an independent predictor of DSS and only well‐established prognostic variables such as tumor size, stage, grade, and surgical treatment influenced outcomes. Despite papillary kidney cancer being three times more common in black patients and a more indolent RCC variant, histology did not appear to influence prognosis. The lack of a racial survival disparity observed contrasts with several previous studies using national registries that demonstrate worse survival for black patients with RCC 10, 12, 14. Paradoxically, we noted that DSS was significantly improved in black patients prior to adjusting for other prognostic variables. This finding is likely driven by the racial differences observed in tumor characteristics in black patients including more favorable tumor stage, lower incidence of metastatic disease, and smaller tumors.

These survival results are consistent with recent study from Lin et al. who evaluated a similar sized cohort of RCC patients (2056 white and 370 black patients) treated at military treatment facilities within the Department of Defense's (DOD) health care system and found no differences in survival by race 19. This consistency with our findings is noteworthy given the differences between our study population and that of Lin et al., which only included active‐duty military personnel, retirees, and dependents. Despite the DOD health care system eliminating many barriers to access to care, patients frequently have supplemental health insurance and may receive some medical services outside the military system. Lin and colleagues also evaluated only overall survival, while we focused on DSS, a much more important measure of biologic aggressiveness. In a population with significant differences in age and comorbidity, an analysis of overall survival alone may mask potential racial differences in disease biology that can influence prognosis.

While our study and Lin's did not demonstrate a racial disparity in survival among black patients with kidney cancer, we must acknowledge that our cohort, despite equivalent access to care, is rather homogenous in many respects compared to other studies that may better reflect the population as a whole. It is widely known that racial disparities exist in employment, health coverage, and socioeconomic status. All black patients in this cohort likely received their healthcare coverage through their employer or their spouse, making them more likely to be in a higher socioeconomic group. We recognize these factors may affect treatment availability and options, something that was not accounted for in our analyses. Socioeconomic differences may largely explain disparities in cancer stage observed among black patients with various forms of malignancy 20. Within RCC, lower socioeconomic status has similarly demonstrated an association with advanced tumor stage 21. Not surprisingly, socioeconomic status significantly influences survival for a wide variety of cancers 22.

Despite evaluating patients within a single healthcare system, among individuals with kidney cancer, there were significant racial differences in both patient and tumor characteristics. The age at diagnosis of kidney cancer was significantly younger in black patients similar to other cohorts 10, 14, 19. We found that papillary RCC was nearly threefold more common in black patients compared to white patients in our cohort. This is consistent with prior single institutional and national registry cohorts that demonstrate a similar increased frequency of papillary histology in black patients 7, 23. Various factors could account for this finding. Specific risk factors for kidney cancer including hypertension, obesity, and chronic kidney disease greatly differ by race 5, 6. Limited studies have evaluated how each of these factors influence the risk of specific histologic types of kidney cancer. Genetic differences may also contribute to a different distribution of histologic subtype. Recently, genome wide association studies (GWAS) have been performed in different racial groups. Interestingly, specific kidney cancer susceptibility loci may differ in the black population 24. Future case–control studies evaluating kidney cancer susceptibility should focus on specific histologic subtypes, as the genomic basis of each differs greatly 25.

The presence of comorbidities with known racial differences such as CKD and hypertension were significantly increased in black patients with kidney cancer. Both of these factors have been shown to increase kidney cancer risk and could explain some of the recent epidemiologic trends in kidney cancer incidence in black patients 5, 6, 26. Additionally, there could be a detection bias in black patients, as often patients with an unknown cause of CKD undergo renal imaging to determine etiology. This practice could lead to an increased incidental detection and skew presentation toward earlier stage disease, resulting in a lead‐time bias. With equal access to healthcare in the Kaiser system and potential early detection of CKD, racial differences in the stage of RCC, perhaps may be even more pronounced than observed in other healthcare systems.

To the best of our knowledge, this is the first study examining cancer‐specific survival disparities in individuals with kidney cancer treated within a single regional healthcare system. We recognize that participation in such a healthcare system does not guarantee equal treatment; however, it can minimize disparate racial access. Another strength is that our data displays the added advantage of having cancer‐specific outcomes especially since other common causes of mortality, including cardiovascular disease, also have racial disparities 27, 28. Unlike studies based only on cancer registry data, we included comorbidity data such as CKD. Inclusion of this may be critical to survival analyses as a recent paper suggests renal comorbidities account for much of the observed racial disparity 29. Besides influencing overall survival, comorbidities may influence specific treatment decisions and similarly affect cancer outcome. Information on systemic therapy was not available at time of recurrence; however, the equivalent rates of surgical treatment may serve as a surrogate of similar treatment at other stages of disease.

## Conclusion

We found that within a large single healthcare system, while some patient and tumor characteristics differed between black and white patients with RCC, surgical treatment rates were similar and race was not an independent predictor of disease‐specific survival. These studies suggest that the disparities in kidney cancer survival outcomes between black and white patients noted in national registries likely result from barriers to access to care rather than disease biology. Future work should focus on identifying, understanding, and then eliminating possible racial barriers to kidney cancer treatment in other healthcare systems.

## Conflicts of Interests

None declared.

## Supporting information


**Table S1.** Univariate analysis of prognostic clinicopathologic variables for disease‐specific survival (DSS) in patients with kidney cancer who received care with Northern California Kaiser Permanente General from 1998 to 2008.
**Table S2.** Demographic, clinical, pathologic, outcomes data of white and black patients with clear cell RCC and received care with Northern California Kaiser Permanente General from 1998 to 2008.
**Table S3.** Demographic, clinical, pathologic, outcomes data of white and black patients with papillary RCC and received care with Northern California Kaiser Permanente General from 1998 to 2008.
