# Supplementary material for: Racial disparities in renal cell carcinoma: a single‐payer healthcare experience
Source: Cancer Med. 2016 May 26;5(8):2101–8. doi: 10.1002/cam4.755 (PMC4884637; doi:10.1002/cam4.755)
Supplement: Supplementary file 1 — Table S1. Univariate analysis of prognostic clinicopathologic variables for disease‐specific survival (DSS) in patients with kidney cancer who received care with Northern California Kaiser Permanente General from 1998 to 2008. Table S2. Demographic, clinical, pathologic, outcomes data of white and black patients with clear cell RCC and received care with Northern California Kaiser Permanente General from 1998 to 2008. Table S3. Demographic, clinical, pathologic, outcomes data of white and black patients with papillary RCC and received care with Northern California Kaiser Permanente General from 1998 to 2008. [file CAM4-5-2101-s001.docx]

**Supplementary Material**

Supplementary Table 1: Univariate analysis of prognostic clinicopathologic variables for Disease Specific Survival (DSS) in patients with kidney cancer who received care with Northern California Kaiser Permanente General from 1998–2008

| Variable | Hazard Ratio (HR) | 95% CI | *p* value |
| --- | --- | --- | --- |
| Age | 1.026 | 1.02–1.03 | <0.001 |
| Gender |  |  | 0.889 |
| Male | 1.00 (ref) |  |  |
| Female | 0.89 | 0.85–1.55 |  |
| Race |  |  | 0.017 |
| White | 1.00 (ref) |  |  |
| Black | 0.73 | 0.57–0.95 |  |
| Smoking History | 1.01 | 0.85–1.20 | 0.914 |
| Hypertension | 1.03 | 0.89–1.20 | 0.683 |
| Diabetes | 0.95 | 0.77–1.17 | 0.607 |
| Anemia | 0.86 | 0.65–1.13 | 0.285 |
| Renal Disease | 0.59 | 0.34–1.02 | 0.060 |
| Tumor size (cm) | 1.04 | 1.04–1.05 | <0.001 |
| Tumor Grade |  |  | <0.001 |
| Grade 1 & 2 | 1.00 (ref) |  |  |
| Grade 3 | 2.92 | 2.27–3.75 | <0.001 |
| Grade 4 | 6.92 | 5.05–9.48 |  |
| Histological Subtype |  |  | 0.364 |
| Clear Cell | 1.00 (ref) |  | <0.001 |
| Papillary | 0.24 | 0.14–0.42 | <0.001 |
| Others | 0.80 | 0.61–1.04 | 0.092 |
| AJCC Stage |  |  | <0.001 |
| Stage 1 & 2 | 1.00 (ref) |  |  |
| Stage 3 & 4 | 15.5 | 12.6–18.9 |  |
| Treatment Status |  |  | <0.001 |
| Surgery | 1.00 (ref) |  |  |
| No Surgery | 12.0 | 10.3–14.1 |  |

Supplementary Table 2: Demographic, clinical, pathologic, outcomes data of white and black patients with clear cell RCC and received care with Northern California Kaiser Permanente General from 1998–2008

| Subcategory | Blacks ccRCC | Whites ccRCC | *p* value |
| --- | --- | --- | --- |
|  | N=208 | N=1829 |  |
| Age |  |  | 0.003 |
| Median | 63 | 66 |  |
| Gender |  |  | 0.008 |
| Male | 113 (54.3%) | 1166 (63.8%) |  |
| Female | 95 (45.7%) | 663 (36.2%) |  |
| BMI* |  |  | 0.824 |
| < 25 | 6 (16.2%) | 75 (19.7%) |  |
| 25 to 30 | 15 (40.5%) | 138 (36.2%) |  |
| >30 | 16 (43.2%) | 168 (44.1%) |  |
| Smoking History |  |  | 0.610 |
| Yes | 54 (26.0%) | 445 (24.3%) |  |
| No | 154 (74.0%) | 1384 (75.7%) |  |
| Hypertension |  |  | <0.001 |
| Yes | 134 (64.4%) | 913 (49.9%) |  |
| No | 74 (34.6%) | 916 (50.1%) |  |
| Diabetes |  |  | 0.072 |
| Yes | 45 (21.6%) | 305 (16.7%) |  |
| No | 163 (78.4%) | 1524 (83.3%) |  |
| CKD |  |  | <0.001 |
| Yes | 20 (9.6%) | 12 (0.7%) |  |
| No | 188 (90.4%) | 1817 (99.3%) |  |
| Any renal disease |  |  | <0.001 |
| Yes | 22 (10.6%) | 42 (2.3%) |  |
| No | 186 (89.4%) | 1787 (97.7%) |  |
| Anemia |  |  | <0.001 |
| Yes | 34 (16.3%) | 153 (8.4%) |  |
| No | 174 (83.7%) | 1676 (91.6%) |  |
| Tumor size (cm) |  |  | 0.118 |
| Median | 4.0 | 5.1 |  |
| Tumor Grade |  |  | 0.396 |
| Grade 1 | 19 (14.7%) | 174 (14.7%) |  |
| Grade 2 | 71 (54.2%) | 558 (47.2%) |  |
| Grade 3 | 35 (26.7%) | 365 (30.9%) |  |
| Grade 4 | 6 (4.6%) | 84 (7.1%) |  |
| T stage |  |  | 0.3833 |
| T1/T2 | 144 (74.6%) | 1197 (71.6%) |  |
| T3/T4 | 49 (25.4%) | 474 (28.4%) |  |
| N Stage |  |  | 0.882 |
| N0/Nx | 203 (97.6%) | 1787 (97.8%) |  |
| N+ | 5 (2.4%) | 41 (2.2%) |  |
| M Stage |  |  | 0.445 |
| M0/Mx | 165 (79.3%) | 1408 (77.0%) |  |
| M+ | 43 (20.7%) | 421 (23.0%) |  |
| AJCC Stage |  |  | 0.347 |
| Stage I/II | 135 (65.2%) | 1123 (61.9%) |  |
| Stage III/IV | 72 (34.8%) | 692 (38.1%) |  |
| Surgery |  |  | 0.173 |
| Yes | 149 (71.6%) | 1390 (76.0%) |  |
| No | 59 (28.4%) | 439 (24.0%) |  |

* BMI data only available for patients enrolled from 2005 - 2008

Supplementary Table 3: Demographic, clinical, pathologic, outcomes data of white and black patients with papillary RCC and received care with Northern California Kaiser Permanente General from 1998–2008

| Subcategory | Blacks papRCC | Whites papRCC | *p* value |
| --- | --- | --- | --- |
|  | N=44 | N=101 |  |
| Age |  |  | 0.003 |
| Median | 57.5 | 64 |  |
| Gender |  |  | 0.528 |
| Male | 32 (72.7%) | 87 (78.4%) |  |
| Female | 12 (27.3%) | 24 (21.6%) |  |
| BMI* |  |  | 0.188 |
| < 25 | 2 (11.1%) | 12 (28.6%) |  |
| 25 to 30 | 7 (38.9%) | 18 (42.9%) |  |
| >30 | 9 (50%) | 12 (28.6%) |  |
| Smoking History |  |  | 0.272 |
| Yes | 20 (45.45%) | 39 (35.1%) |  |
| No | 24 (54.55%) | 72 (64.9%) |  |
| Hypertension |  |  | 0.095 |
| Yes | 33 (75%) | 66 (59.5%) |  |
| No | 11 (25%) | 45 (40.5%) |  |
| Diabetes |  |  | 0.595 |
| Yes | 7 (15.9%) | 13 (11.7%) |  |
| No | 37 (84.1%) | 98 (88.3%) |  |
| CKD |  |  | 0.006 |
| Yes | 7 (15.9%) | 3 (2.7%) |  |
| No | 37 (84.1%) | 108 (97.3%) |  |
| Any renal disease |  |  | 0.002 |
| Yes | 9 (20.45%) | 4 (3.6%) |  |
| No | 35 (79.55%) | 107 (96.4%) |  |
| Anemia |  |  | 0.088 |
| Yes | 8 (18.2%) | 9 (8.1%) |  |
| No | 36 (81.2%) | 102 (91.9%) |  |
| Tumor size |  |  | 0.063 |
| Median | 5.35 | 4.2 |  |
| Tumor Grade |  |  | 0.311 |
| Grade 1 | 7 (21.2%) | 12 (15.8%) |  |
| Grade 2 | 14 (42.4%) | 45 (59.2%) |  |
| Grade 3 | 12 (36.4%) | 18 (23.7%) |  |
| Grade 4 | 0 (0%) | 1 (1.3%) |  |
| T stage |  |  | 1.000 |
| T1/T2 | 38 (88.4%) | 97 (89%) |  |
| T3/T4 | 5 (11.6%) | 12 (11%) |  |
| N Stage |  |  | 0.319 |
| N0/Nx | 42 (95.5%) | 109 (98.2%) |  |
| N+ | 2 (4.5%) | 2 (1.8%) |  |
| M Stage |  |  | 0.676 |
| M0/Mx | 43 (97.7%) | 106 (95.5%) |  |
| M+ | 1 (2.3%) | 5 (4.5%) |  |
| AJCC Stage |  |  | 1.000 |
| Stage I/II | 37 (84.1%) | 93 (83.8%) |  |
| Stage III/IV | 7 (15.9%) | 18 (16.2%) |  |
| Surgery |  |  | 1.000 |
| Yes | 42 (95.5%) | 105 (94.6%) |  |
| No | 2 (4.5%) | 6 (5.4%) |  |

* BMI data only available for patients enrolled from 2005 - 2008
